# Neuroinflammation and blood-brain barrier breakdown in acute, clinical intracerebral hemorrhage

**DOI:** 10.1177/0271678X241274685

**Published:** 2024-10-03

**Authors:** Olivia A Jones, Saffwan Mohamed, Rainer Hinz, Alastair Paterson, Oluwaseun A Sobowale, Ben R Dickie, Laura M Parkes, Adrian R Parry-Jones

**Affiliations:** 1 Division of Psychology, Communication and Human Neuroscience, School of Health Sciences, Faculty of Biology, Medicine and Health, University of Manchester, Manchester, UK; 2 Geoffrey Jefferson Brain Research Centre, Faculty of Biology, Medicine and Health, University of Manchester, Manchester, UK; 3 Division of Cardiovascular Sciences, School of Medical Sciences, Faculty of Biology, Medicine and Health, University of Manchester, Manchester, UK; 4 Division of Imaging, Informatics and Data Sciences, School of Health Sciences, Faculty of Biology, Medicine and Health, University of Manchester, Manchester, UK; 5 Manchester Centre for Clinical Neurosciences, Northern Care Alliance NHS Foundation Trust, Salford, UK

**Keywords:** Intracerebral hemorrhage, microglial activation, blood-brain barrier, DCE-MRI, PK11195 PET

## Abstract

Neuroinflammation is a promising therapeutic target in intracerebral hemorrhage (ICH), characterized in the brain by microglial activation and blood-brain barrier (BBB) breakdown. In this study, 36 acute, spontaneous, supratentorial ICH patients underwent dynamic contrast-enhanced MRI to measure BBB permeability (*K*^trans^) 1–3 days post-onset and 16 returned for [^11^C](*R*)-PK11195 PET to quantify microglial activation (*BP_ND_*), 2–7 days post-onset. We first tested if these markers were increased and co-localized in the perihematomal brain and found that perihematomal *K*^trans^ and *BP_ND_* were increased vs. the contralateral brain, but regions of high *K*^trans^ and *BP_ND_* only overlapped by a mean of 4.9%. We then tested for associations of perihematomal *K*^trans^ and *BP_ND_* with clinical characteristics (age, ICH volume & location, blood pressure), other markers of inflammation (edema, IL-6, and CRP), and long-term functional outcome (90-day mRS). Lower perihematomal *BP_ND_* was associated with increasing age. Lobar hemorrhage was associated with greater *K*^trans^ than deep, but *K*^trans^ and *BP_ND_* were not associated with ICH volume, or other inflammatory markers. While perihematomal *K*^trans^ and *BP_ND_* were not associated with outcome, contralateral *K*^trans^ was significantly associated with greater 90-day mRS. Exploratory analyses demonstrated that blood pressure variability over 72 h was also associated with contralateral *K*^trans^.

## Introduction

On a global scale, spontaneous intracerebral hemorrhage (ICH) accounts for 28% of incident strokes, 44% of the 6.55 million stroke deaths annually, 48% of the disability burden created by stroke,^1^ and has relatively few effective treatments.^2^ Reversal of anticoagulants and intensive blood pressure lowering help to reduce the risk of hematoma expansion,^3^ and surgery may be offered for a carefully selected minority, often as a lifesaving procedure.^2,4^ Aside from good supportive care, there is little to offer most patients. Given that most ICH occurs in low to middle income countries^1^ and the high cost associated with surgery and critical care, there remains a clear unmet need for a simple, cheap, and effective treatment that improves outcomes for most patients. Targeting the secondary injury resulting from ICH may help to realize this ambition.

ICH is characterized by immediate physical injury followed by a secondary cascade of pathophysiological changes.^5^ Tissue injury markers and components of extravasated blood act as damage-associated molecular patterns, which are detected by pattern-recognition receptors on the surface of ramified microglia. These microglia become activated, expressing a proinflammatory phenotype within hours of onset, releasing cytokines and chemokines. These activate astrocytes and endothelial cells thought to cause blood-brain barrier (BBB) breakdown, recruitment of leukocytes, and perihematomal edema formation.^6^ Ongoing tissue injury occurs due to breakdown of blood products such as hemoglobin, heme and iron.^7^ From around 72 h, there is a gradual switch of the inflammatory response towards a repair and recovery phenotype.^5^ Inhibiting the early, damaging pro-inflammatory response and/or enhancing the later repair and recovery functions of inflammation represent promising treatment targets with an extended therapeutic window.

Our understanding of the inflammatory response to ICH is largely derived from experimental models that do not fully recapitulate the clinical disease.^8^ The time course of inflammation may be more prolonged in the clinical disease, where edema peaks at 1–2 weeks^9^ and persists for up to 1–2 months, and the hematoma may take months to clear.^10^ In experimental ICH, both the edema and the hematoma have generally cleared by one week.^11^ *In vivo* clinical studies are limited by access to samples and often rely on simple measures such as edema on CT scans^12^ or measurement of inflammatory markers in venous blood^13^ as surrogate measures of the inflammatory response. Advanced neuroimaging methods can non-invasively quantify inflammation in the human brain after injury. Specifically, [^11^C](*R*)-PK11195 PET maps the translocator protein 18 kDa (TSPO) expressed on activated microglia and macrophages. Dynamic contrast enhanced (DCE)-MRI tracks the movement of contrast agent through and out of leaky vessels to map BBB permeability. PET imaging is especially challenging in acute ICH patients, but our previous pilot study demonstrated the feasibility of performing PET combined with DCE-MRI in a small group of acute ICH patients.^14^

Here, we report results of a study applying these methods to a larger cohort of acute ICH patients with the following aims:

1. Determine whether BBB permeability and TSPO binding are elevated in the perihematomal region and if so, describe extent to which these co-localize, as this has clear implications for the delivery of peripherally administered immunomodulatory treatments to activated immune cells in acute ICH.
2. Test whether perihematomal edema and circulating inflammatory markers are associated with BBB permeability and TSPO binding, improving our understanding of the use of perihematomal edema and peripheral biomarkers commonly used as surrogate markers of brain inflammation after ICH.
3. Test for associations between key clinical characteristics (age, ICH volume and location, systolic blood pressure) and BBB permeability and TSPO binding, which may reveal subgroups where brain inflammation is more pronounced.
4. Determine whether BBB breakdown and TSPO binding are associated with long-term functional outcome.

## Materials and methods

### Participants

This study was conducted in full conformity with the current revision of the Declaration of Helsinki, with approvals obtained from the Health Research Authority (IRAS ID: 181847), and the study was registered with the ISRCTN (Ref: 52682983). Patients with acute, spontaneous, supratentorial ICH were recruited from Salford Royal Hospital (Salford, UK) between 19/12/2015 and 01/05/2018. Written informed consent was obtained from all patients. Patients within 24 hours of symptom onset, a Glasgow Coma Scale (GCS) score > 9, who could undergo MRI within 72 hours of recruitment were included. Exclusion criteria were a suspected or confirmed secondary cause of ICH, contraindication to MRI, pre-existing brain lesions such as previous stroke or tumor, history of recent head trauma, known inflammatory disease, uncorrected bleeding disorder, pregnancy or breast feeding, estimated glomerular filtration rate <30 ml/min/1.73 m^2^, neurosurgical procedure performed or planned, or treatment with drugs known to interfere with PK-11195 binding (benzodiazepines, steroids, minocycline). Only patients deemed medically stable, with a GCS > 12, were included in the PET imaging component of this study. Clinical data were collected at baseline and at each scan time point and included age, pre-ICH hypertension, blood pressure recordings at baseline, 2 h, 4 h, 6 h, 12 h, 24 h, 48 h and 72 h blood pressure. The mean and standard deviation of the systolic blood pressure (SBP) was calculated using all available blood pressure measurements from 2 to 72 h.

### Structural imaging

MRI data were acquired on a Philips 3 T Achieva scanner (Salford Royal Hospital) with an 8-channel head coil. Structural imaging consisted of a 3D T_1_-weighted image with a resolution of 0.94 mm ×0.94 mm × 1 mm, and an acquisition time of 311 s. For assessment of perihematomal edema, a T_2_-weighted FLAIR image was acquired with an in-plane resolution of 0.69 mm × 0.69 mm, 100 contiguous axial slices of 1.3 mm thickness, and an acquisition time of 250 s. ICH location was classified as deep or lobar using the CHARTS instrument.^15^

### Dynamic contrast-enhanced MRI

Quantities, processes and model definitions are OSIPI CAPLEX compliant.^16^ CAPLEX definitions can be accessed by clicking on quantity, process or model hyperlinks. A dynamic series of 3D T_1_-weighted Fast Field Echo (T_1_-FFE; spoiled gradient recalled echo) images were acquired with a repetition time of 2.4 ms, echo time of 0.8 ms, prescribed excitatory flip angle of 10 degrees, an in-plane resolution of 1.5 mm × 1.5 mm, 32 contiguous axial slices of 4 mm thickness, and an acquisition time of 7.6 s for each dynamic. Eighty dynamic images were acquired over 10 minutes. On the 8th dynamic, a gadolinium-based contrast agent (Gd-DOTA; Dotarem, Guebert, France) bolus was administered using a power injector at a dose of 0.1 mmol/kg.

Prior to the dynamic scan, a series of additional 3D T_1_-FFE images were acquired at 4 different excitatory flip angles (2, 5, 10, and 15 degrees) to calculate the pre-contrast (native) longitudinal relaxation rate, R_10_. Acquisition parameters remained consistent with the dynamic scan except that only eight signal averages were acquired, giving a total acquisition time of 60 s per flip angle. To correct R_10_ for B1 field inhomogeneities, a pair of B1 mapping images were also acquired with the same voxel size and coverage as the variable flip angle images. This consisted of a pair of 3D T_1_-FFE images with repetition times of 25 ms and 125 ms, respectively, echo time of 5 ms, a flip angle of 60 degrees, and an acquisition time of 117 s.

The DCE-MRI data was analyzed as described in the supplementary materials; briefly, the B_1_-corrected R_10_ map was calculated by fitting the spoiled gradient recalled echo signal model to signal from variable flip angles. The resulting R_10_ map used to estimate contrast agent concentration from the dynamic series of T_1_-weighted images. A Patlak model of contrast agent uptake, with an input function derived from a region drawn in the superior sagittal sinus, was fit to the concentration time course on a voxel-wise basis to produce maps of the contrast agent volume transfer constant, *K* ^trans^ .

### PET imaging

PET scans took place on a high-resolution research tomograph (HRRT; Siemens/CTI). A 7-minute transmission scan with a ^137^Cs point source for subsequent attenuation and scatter correction was followed by a 60-minute emission scan (one background frame of approximately 7 minutes, prior to injection, followed by 17 frames: 1 × 15 s, 1 × 5 s, 1 × 10 s, 1 × 30 s, 4 × 60 s, 7 × 300 s, and 2 × 600 s). A slow bolus of [^11^C](*R*)-PK11195 was injected intravenously over 15 s at the beginning of the emission scan. Across the cohort, a mean activity of 711 ± 65 MBq was injected, with a mean of 1.5 ± 1.2 μg of unlabeled PK11195. PET images were reconstructed with the iterative ordinary Poisson ordered-subset maximization algorithm (16 subsets, 12 iterations) using isotropic voxels of 1.2 mm^3^ and were smoothed post reconstruction with a 3-D Gaussian filter (full width at half maximum = 4 mm). The simplified reference tissue model was used with a reference tissue input function from the cerebellar grey matter to generate parametric maps of binding potential, *BP_ND_*.

### Region of interest analysis

Regions corresponding to the hematoma and perihematomal edema were manually outlined on the T_2_-weighted FLAIR image by a Neurosurgical Research Fellow (SM) and used to calculate the ICH volume and edema extension distance (EED).^17^ The hematoma and perihematomal regions were reflected about the midline in the axial plane to generate size-matched contralateral regions, sufficiently remote from the hematoma to act as a representative region for the assessment of global brain changes. The perihematomal region was dilated using the imdilate function in Matlab (Version 2022 b, Mathworks, Natick, Massachusetts) to produce twenty-five concentric shells around the perihematomal region, each with a thickness of 2 mm. All regions of interest and parametric maps were co-registered to the high-resolution 3D T_1_-weighted structural image using the co-registration function in SPM12.

Median values for *K*^trans^ and *BP_ND_* were extracted from the perihematomal, contralateral, and dilated regions. To quantify co-localization between BBB permeability and TSPO binding, patient-specific thresholds were computed, defined as two standard deviations above the contralateral median. Perihematomal voxels above this threshold were defined as having significantly elevated perihematomal *K*^trans^ and *BP_ND_*, and the overlap of these voxels as a percentage of the high *BP_ND_* region was calculated.

### Peripheral inflammatory markers

At the time of MR and PET scanning, respectively, approximately 6 ml of venous blood was taken for immunoassays. Measurement of IL-6 was by enzyme linked immunosorbent assay and plasma CRP by single plex competitive assay, as detailed in the supplementary materials.

### Clinical outcome measures

Participants underwent clinical follow-up at 90 days (+/− 7 days) post-stroke with an experienced Stroke Neurologist (APJ) where the modified Rankin Scale (mRS) score was determined.

### Statistical analysis

Statistical analyses were performed in GraphPad Prism (version 8.0.0, GraphPad Software, San Diego, USA) and IBM SPSS Statistics (Version 29, SPSS Inc., Chicago, USA). To address Aim 1, paired *t*-tests were performed to compare median values for *K*^trans^ and *BP_ND_* between the perihematomal and contralateral regions. Pearson’s correlation tests were used to test for association between perihematomal *K*^trans^ and *BP_ND_*, as well as between the volume of elevated *K*^trans^ and *BP_ND_* voxels in the perihematomal region.

To address Aims 2 and 3, multifactorial linear regression analyses were performed to test for associations with inflammatory markers and baseline clinical characteristics. These consisted of two multifactorial models: firstly, with perihematomal *K*^trans^ as the dependent variable, with CRP, IL-6, EED, location (deep/lobar), time since onset, age, ICH volume, mean SBP, and SD SBP as covariates. This was repeated with perihematomal *BP_ND_* as the dependent variable. CRP, IL-6 and ICH volume were log transformed prior to statistical analysis to achieve a normal distribution.

To address Aim 4, multifactorial linear regression analyses were performed to test for associations between imaging measures of inflammation and outcome. Firstly, with 90-day mRS as the dependent variable and perihematomal *K*^trans^ as a covariate. Other factors with the potential to influence relationships between imaging markers and outcome were also included as covariates: ICH location, time since onset, age, ICH volume, mean and SD SBP. Contralateral *K*^trans^ was also included in the model as a covariate to correct for potential whole-brain inflammatory changes. This was repeated with perihematomal and contralateral *BP_ND_* as covariates. All statistical tests had an *α* value of 0.05.

## Results

### Participants

Forty-four patients were recruited, of whom 40 underwent DCE-MRI scans 1–3 days after onset. Four patients did not complete the full DCE-MRI scan and the remaining 36 were scanned at a median of 56.2 h (IQR 43.9 to 66.4 h; range 26.1 to 87.3 h) after onset. Sixteen participants returned for a dynamic PET scan, 3–8 days after onset (median onset-to-PET scan time was 113.5 h, IQR 94.2 to 134.3 h, range 59.7 to 194.4 h). For the subset of patients having MR and PET, the median interval between scans was 67.8 h (IQR 39.6 to 73.8 h). Baseline characteristics, imaging findings, inflammatory marker results, and long-term outcomes are shown in Table 1. There was no difference between ICH volumes (*P = *0.2), GCS (*P = *0.9) or NIHSS scores (*P = *0.5) between the two groups. Three participants had missing data for inflammatory markers at the time of MR scan. Two participants were lost to follow-up.

**Table 1.** Summary characteristics and measures from the cohort, divided into those who underwent MRI only and those who underwent both PET and MR imaging.

Characteristic	Level	MR Only (n = 20)	PET and MR (n = 16)
Age	Mean (SD)	69 (13)	67 (12)
ICH volume (ml)	Median (IQR)	19.9 (8.4, 34.7)	38.7 (18.2, 43.7)
	Minimum–Maximum	2.9–78.5	5.7–54.6
PHE volume (ml)	Median (IQR)	5.9 (2.2, 11.7)	10.7 (6.1, 17.4)
	Minimum–Maximum	0.6–20.4	0.8–28.5
NIHSS on admission	Median (IQR)	6 (4, 8)	8 (5–13)
	Minimum–Maximum	1–23	1–23
Glasgow Coma Scale Score	Median (IQR)	15 (15, 15)	15 (15, 15)
	Minimum–Maximum	10–15	12–15
Sex			
Female	N (%)	5 (33%)	8 (50%)
Male	N (%)	15 (67%)	8 (50%)
Risk factors			
Hypertension	N (%)	14 (70%)	7 (44%)
Atrial fibrillation	N (%)	1 (5%)	3 (19%)
Diabetes	N (%)	2 (10%)	0 (0%)
SBP			
Baseline	Mean (SD)	178.9 (38.8)	171.6 (24.7)
Mean Over 72 h	Mean (SD)	142.3 (12.7)	142.0 (11.0)
SD Over 72 h	Mean (SD)	16.0 (12.7)	13.0 (6.2)
Time from onset to scan			
MR (hours)	Median (IQR)	63.8 (42.0, 75.6)	54.3 (44.8, 61.1)
	Minimum–Maximum	26.1–87.3	35.7–77.0
PET (hours)	Median (IQR)	–	113.5 (94.2, 134.3)
	Minimum–Maximum		59.7–194.4
ICH location			
Deep	N (%)	10 (50%)	11 (69%)
Lobar	N (%)	10 (50%)	5 (31%)
CAA classification			
No CAA	N (%)	13 (65%)	12 (75%)
Possible CAA	N (%)	4 (20%)	3 (19%)
Probable CAA	N (%)	3 (15%)	1 (6%)
IL-6 (pg/ml)			
On date of MR	Median (IQR)	16.7 (6.9, 30.3)	3.6 (1.8–4.9)
	Minimum–Maximum	0.3–69.8	0.1–41.9
On date of PET	Median (IQR)	–	3.9 (2.0–8.1)
	Minimum–Maximum		0.1–28.0
CRP (mg/l)			
On date of MR	Median (IQR)	4.7 (2.8–8.4)	6.5 (2.9–7.8)
	Minimum–Maximum	0.3–45.3	0.2–17.7
On date of PET	Median (IQR)	–	7.1 (4.8–12.1)
	Minimum–Maximum		−0.7–57.5
Imaging metrics			
Ipsilateral *K^trans^* (×10^−3 ^min^−1^)	Mean (SD)	0.94 (0.42)	0.76 (0.23)
Contralateral *K^trans^* (×10^−3 ^min^−1^)	Mean (SD)	0.49 (0.39)	0.45 (0.20)
Ipsilateral *BP_ND_*	Mean (SD)	–	0.14 (0.34)
Contralateral *BP_ND_*	Mean (SD)	–	0.02 (0.23)
Overlap between elevated *K^trans^* and *BP_ND_ (%)*	Mean (SD)	–	4.9 (5.9)
90-day mRS			
1	N (%)	2 (10%)	4 (27%)
2	N (%)	8 (40%)	3 (20%)
3	N (%)	4 (20%)	5 (33%)
4	N (%)	2 (10%)	1 (7%)
5	N (%)	2 (10%)	1 (7%)
6	N (%)	1 (5%)	1 (7%)

ICH: intracerebral hemorrhage; PHE: perihematomal edema; NIHSS: National Institutes of Health Stroke Scale; CAA: cerebral amyloid angiopathy; IL-6: interleukin 6; CRP: C-reactive protein; mRS: modified Rankin Scale score; SBP: systolic blood pressure.

### Co-localization of BBB breakdown and TSPO binding

**Figure 1.**
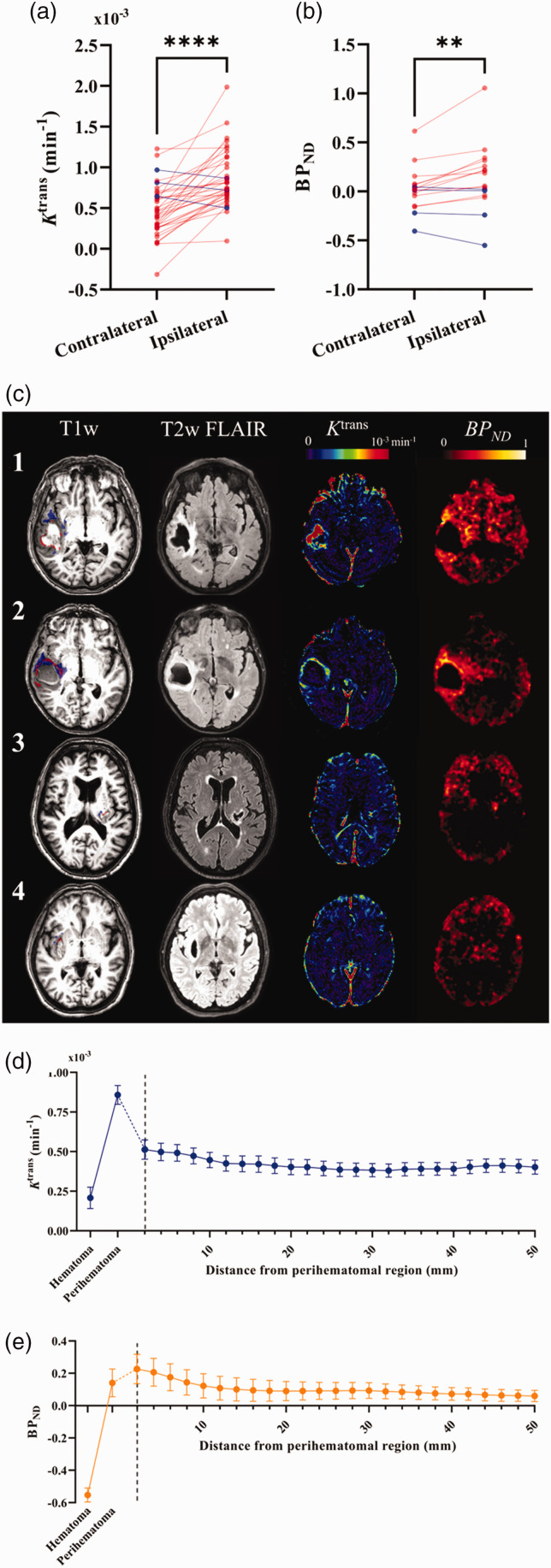
Spatial patterns of neuroinflammation after ICH. (a–b) Individual values of median imaging metrics in the ipsilateral (perihematomal) and contralateral regions are presented for (a) *K*^trans^ and (b) *BP_ND_*. Red datapoints represent increased values in the ipsilateral region compared to the contralateral region, blue represents a decrease. Statistics shown correspond to results of Continued.a paired t-test where **** represents *P* < 0.0001 and **P < 0.01. (c) A high-resolution T_1_-weighted image overlayed with regions of high *K*^trans^ (red) and high [^11^C](*R*)-PK11195 binding potential (blue), T_2_-weighted FLAIR, DCE-MRI *K*^trans^ map, and [^11^C](*R*)-PK11195 binding potential (*BP_ND_*) map are shown for a representative 4 (of 16) patients that underwent both [^11^C](*R*)-PK11195 PET and DCE-MRI scans 1–7 days post-ICH. 1 – A 72-year-old male patient with a 45 ml lobar ICH. MR performed at 48 h and PET at 116 h after onset. 2 – An 81-year-old female patient with a 54 ml lobar ICH. MR performed at 74 h and PET at 148 h after onset. 3 – A 75-year-old male patient with a 6 ml deep ICH. MR performed at 49 h and PET at 190 h after onset. 4 – A 66-year-old female patient with a 10 ml deep ICH. MR performed at 36 h and PET at 109 h after onset. (d–e) Plots of mean *K*^trans^ (d) and *BP_ND_* (e) across the cohort from concentric spherical shells of 2 mm thickness spreading from the perihematomal edema. Mean values across the cohort for the hematoma and perihematomal region are also shown for reference. Error bars shown are the standard error of the mean.

Median *K*^trans^ and *BP_ND_* were greater in the perihematomal region compared to the contralateral region (Figure 1(a) and 1(b); *P < *0.0001 and *P = *0.006, respectively). Maps of *K*^trans^ and *BP_ND_* are shown for four representative patients in Figure 1(c). BBB breakdown was identified around the perihematomal rim for all cases, though the distribution and intensity varied. Figure 1(d) displays median *K*^trans^ values in the perihematomal region and concentric rings spreading out from the region, averaged across patients, showing a sharp peak in the intensity of *K*^trans^ in the perihematomal region. Moving further away from the lesion a relatively constant, lower intensity was observed across the concentric regions. Spatial patterns of TSPO binding were varied and, in some patients, a widespread inflammatory response was observed on *BP_ND_* maps (e.g. Figure 1(c), Patient 1), whereas in others it was localized to the immediate perihematomal region (e.g. Figure 1(c), Patient 3). Across the group, the intensity of median *BP_ND_* was greatest outside the perihematomal region, peaking at 0–2 mm beyond the lesion, with a gradual decrease in intensity moving further from the lesion (Figure 1(e)).

**Figure 2.**
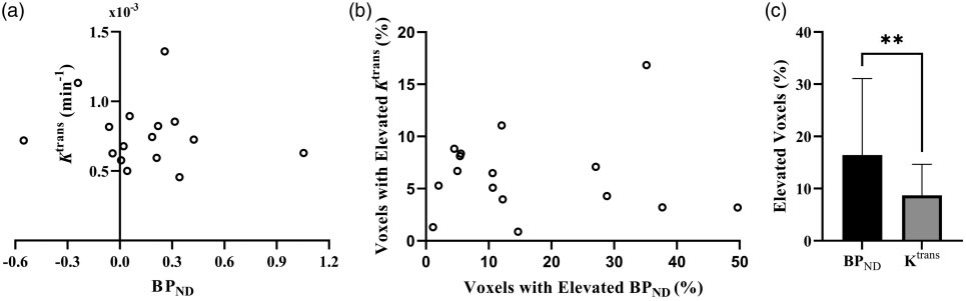
Correlation between immune cell activation and BBB permeability. (a) Perihematomal *K*^trans^ plotted against perihematomal *BP_ND_*. (b) Volume of elevated *K*^trans^ as a percentage of the perihematomal edema volume plotted against the percentage volume of elevated *BP_ND_* and (c) Mean values for the volume of elevated *K*^trans^ and *BP_ND_* as percentages of the perihematomal edema volume. Error bars shown are the standard deviation of the mean.

There was no association between perihematomal *K*^trans^ and *BP_ND_* (Figure 2(a)), nor was the volume of elevated *K*^trans^ associated with the volume of elevated *BP_ND_* in the perihematomal region (Figure 2(b)). The volume of elevated *BP_ND_* was greater than the volume of elevated *K*^trans^ in the perihematomal region, further suggesting that increases in perihematomal TSPO binding are more spatially extensive than BBB permeability (*P = *0.009; Figure 2(c)). Voxels with elevated *K*^trans^ and *BP_ND_* showed little overlap, with a mean of 4.9% (SD 5.9%) of the elevated *BP_ND_* voxels also having elevated *K*^trans^.

### Factors associated with BBB breakdown and TSPO binding

**Figure 3.**
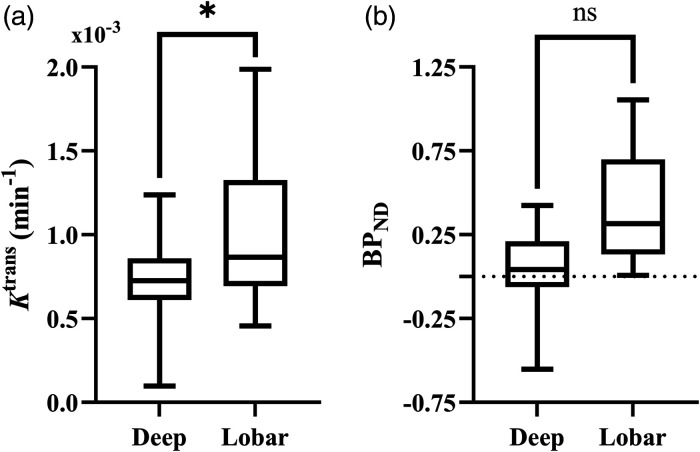
Comparison of immune cell activation and BBB permeability in deep and lobar hemorrhage. Box plot comparing distribution of perihematomal (a) *K*^trans^ and (b) *BP_ND_* in deep and lobar hemorrhage. Statistical significance in the multifactorial regression correcting for other clinical and inflammatory factors are indicated, where * represents *P* < 0.05 and ns *P* ≥ 0.05.

Perihematomal imaging markers for BBB permeability and TSPO binding were not associated with ICH volume, mean or SD SBP, or time from onset to scan (MR or PET, respectively). Higher perihematomal *BP_ND_* was associated with lower age (ß = −0.264, *P = *0.04) however *K*^trans^ was not associated. Perihematomal *K^trans^* and *BP_ND_* were not correlated with circulating inflammatory markers (IL-6 and CRP) measured at the MR scan and PET scan, respectively. Higher *K*^trans^ was found to be associated with lower EED (ß = −0.581, *P = *0.03), while *BP_ND_* was not associated with EED. *K*^trans^ was associated with ICH location (ß = −0.491, *P = *0.01), where patients with lobar ICH had a higher perihematomal *K*^trans^ than those with deep ICH (Figure 3), however associations between *BP_ND_* and location were not significant. Coefficients and *P* values for the multifactorial regression analyses are presented in Table 2.

**Table 2.** Association between imaging metrics, factors relating to inflammation, and clinical characteristics.

Measure	*K* ^trans^		*BP_ND_*	
	*β coefficient (95% CI)*	*p*	*β coefficient (95% CI)*	*p*
CRP (mg/L)	0.149 (−0.043 to 0.111)	0.37	0.107 (−0.045 to 0.097)	0.3
IL-6 (pg/mL)	0.006 (−0.105 to 0.108)	0.97	−0.150 (−0.139 to 0.040)	0.2
EED (mm)	−0.581 (−0.218 to −0.014)	0.03*	−0.316 (−0.143 to 0.010)	0.07
Location (Deep)	−0.491 (−0.617 to −0.088)	0.01*	−0.264 (−0.015 to −0.001)	0.06
Time since onset (days)	0.112 (−0.005 to 0.010)	0.46	−0.110 (−0.073 to 0.027)	0.2
Age (Years)	−0.125 (−0.014 to 0.007)	0.48	−0.264 (−0.025 to −0.001)	0.04*
ICH Volume (mm^3^)	0.535 (−0.023 to 0.395)	0.08	0.344 (−0.006 to 0.261)	0.06
Mean SBP (mm Hg)	−0.190 (−0.16 to 0.005)	0.27	0.048 (−0.005 to 0.008)	0.5
SD SBP (mm Hg)	0.183 (−0.010 to 0.025)	0.38	−0.012 (−0.020 to 0.019)	0.9

ICH: intracerebral hemorrhage; SBP: systolic blood pressure; EED: edema extension distance; IL-6: interleukin 6; CRP: C-reactive protein.

### BBB breakdown, TSPO binding and outcome

**Figure 4.**
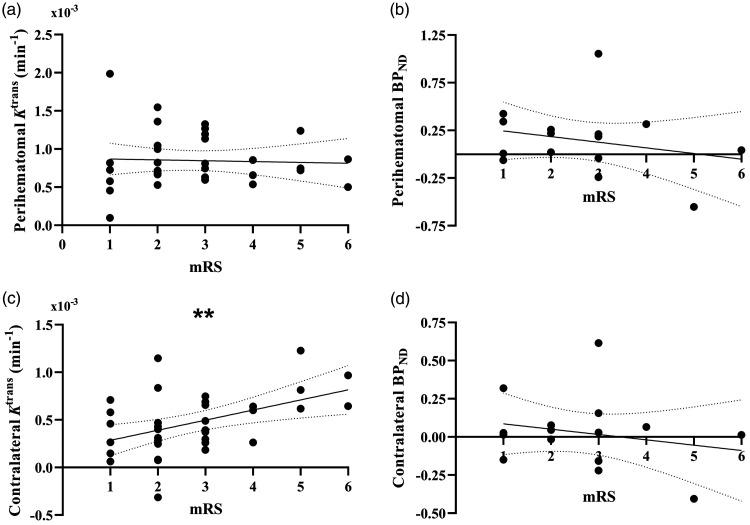
Correlation between imaging biomarkers and functional outcome. Plots of perihematomal (a) *K*^trans^ and (b) *BP_ND_* against modified Rankin Scale scores (mRS), as well as contralateral (c) *K*^trans^ and (d) *BP_ND_* against mRS. Lines of best fit and 95% CI bands from linear regression analysis are shown for each graph. Statistical significance is indicated where ** represents *P* < 0.01.

Outcomes of multifactorial regression analyses testing associations between outcome and imaging measures are presented in Table 3. After adjustment for factors thought to influence relationships with outcome (contralateral *K*^trans^/*BP_ND_*, ICH location, time from onset to scan, age, ICH volume, mean and SD SBP) neither perihematomal *K*^trans^ nor *BP_ND_* were associated with mRS at 90 days. Figure 4(a) and (b) visualize absolute *K*^trans^ and *BP_ND_* values in the perihematomal region against 90-day mRS. Conversely, contralateral *K*^trans^ was found to be significantly greater with higher mRS (ß = 0.492, *P = *0.009), presented in Figure 4(c).

**Table 3.** Association between outcome and imaging metrics.

Measure	*mRS*		Measure	*mRS*	
	*β coefficient (95% CI)*	*p*		*β coefficient (95% CI)*	*p*
Perihematomal *K*^trans^	−0.355 (−2.805 to 0.040)	0.06	Perihematomal *BP_ND_*	0.460 (−13.020 to 16.904)	0.8
Contralateral *K*^trans^	0.492 (0.602 to 3.725)	0.009*	Contralateral *BP_ND_*	−0.503 (−21.155 to 14.731)	0.7
Location (Deep)	0.245 (−0.401 to 1.788)	0.2	Location (Deep)	0.675 (−4.241 to 8.378)	0.5
Time since onset (days)	−0.053 (−0.032 to 0.022)	0.7	Time since onset (days)	−0.481 (−1.130 to 0.316)	0.3
Age (Years)	0.111 (−0.025 to 0.050)	0.5	Age (Years)	0.426 (−0.139 to 0.243)	0.5
ICH Volume (mm^3^)	0.203 (−0.234 to 0.771)	0.3	ICH Volume (mm^3^)	0.234 (−0.985 to 1.691)	0.5
Mean SBP (mm Hg)	0.373 (0.006 to 0.078)	0.02*	Mean SBP (mm Hg)	0.510 (−0.051 to 0.180)	0.2
SD SBP (mm Hg)	0.211 (−0.034 to 0.100)	0.3	SD SBP (mm Hg)	0.103 (−0.227 to 0.279)	0.8

ICH: intracerebral hemorrhage; SBP: systolic blood pressure; mRS: modified Rankin Scale score.

As an additional post-hoc analysis to evaluate whether contralateral *K*^trans^ is truly elevated in patients with worse outcome, we tested whether patients with contralateral *K*^trans^ greater than the 95% confidence interval of control grey matter *K*^trans^ from another study^18^ had worse outcomes using an unpaired t-test, finding that patients with contralateral *K*^trans^ greater than that of controls had significantly worse mRS scores (3.29 vs. 2.23, *P = *0.007), shown in Supplementary Figure 1. To further probe this result and generate future hypotheses, we then used a linear regression model to investigate whether baseline and ICH-specific measures were associated with contralateral *K*^trans^, the results of which are shown in Supplementary Table 1. ICH location, time from onset to scan, age, ICH volume, and mean SBP over the first 72 h were not associated with contralateral *K*^trans^, however the SD of SBP in the first 72 h was associated with contralateral *K*^trans^ (ß = 0.507, *P = *0.04).

## Discussion

Using DCE-MRI and [^11^C](*R*)-PK11195 PET to measure BBB permeability and TSPO binding in a cohort of acute ICH patients, we show that both BBB permeability and TSPO binding are greater in the perihematomal brain compared to the contralateral hemisphere. We found no correlation between the magnitude of BBB permeability and TSPO binding in the perihematomal edema and little co-localization of voxels where these measures were elevated. We found perihematomal BBB permeability to be significantly greater in lobar hemorrhage compared with deep locations. TSPO binding was greater with younger age. We found a significant inverse association between BBB permeability and edema, but circulating inflammatory markers were not associated with perihematomal BBB permeability or TSPO binding. Finally, we show that greater BBB permeability in the contralateral brain 1–3 days after onset was associated with poor outcome at 90 days, but there was no association between TSPO binding 2–8 days after onset and outcome. Further exploratory analyses to understand the association between contralateral *K*^trans^ and 90-day mRS suggest that increased variation in SBP over 72 h is associated with greater contralateral BBB permeability. Very few human studies have measured neuroinflammation *in vivo* and tested for associations with clinical outcomes, and aside from our prior pilot study, none have studied ligands for TSPO in clinical ICH.^19^ Thus, the current study provides unique insights into neuroinflammation after ICH in humans.

We found that the volume of elevated TSPO binding was greater than that of BBB dysfunction. Regions of elevated BBB breakdown were observed around the rim of the hematoma, as observed in previous studies,^14,20^ whereas elevated TSPO binding had more dispersed patterns. The lack of age and risk-factor matched controls limits our ability to interpret the widespread TSPO binding signal observed remotely from the hematoma in some patients (Figure 1(c)). We found little overlap between voxels with elevated BBB permeability and TSPO binding and found that the magnitude and volume of BBB breakdown and TSPO binding in the perihematomal edema did not correlate. Similar results have been reported in patients with sporadic and monogenic chronic small vessel disease, suggesting that this observation may not be restricted to acute cerebrovascular disease.^21^ This has important implications for the delivery of immune modulating therapies, which may not be able to rely on increased BBB permeability to penetrate into the brain, at least at 1–3 days post-onset. Treatments capable of crossing an intact BBB or direct delivery to the hematoma cavity at the time of surgery may be needed.

BBB permeability was negatively associated with edema, indicating that greater perihematomal *K*^trans^ was related to smaller EED. This result is surprising, and in contrast a previous study found DCE-MRI measures of BBB permeability to be positively correlated with edema volume at both one day and one week after ICH.^20^ As observed on parametric maps of *K*^trans^ (e.g. Figure 1(c)), regions of high BBB permeability tended to be close to the hematoma rather than filling the edema, meaning *K*^trans^ values extracted from larger perihematomal edema regions may be somewhat diluted, leading to the negative association found via multiple regression analysis. It is also possible that early edema may be driven by accumulation of serum in the brain parenchyma when extravasated blood clots,^22^ and this process is independent of the BBB. A previous study found no association between BBB permeability at baseline and edema growth at 24 h.^23^ The lack of any association between EED and TSPO binding suggests that caution should be applied when using the EED as a surrogate measure of the inflammatory response at day 1–3. Similarly, circulating IL-6 and CRP at the time of the MR and PET scans showed no correlation with BBB permeability or TSPO binding.

Lobar hemorrhages were associated with greater BBB permeability than deep. Greater BBB permeability in hemorrhages in lobar locations has been reported elsewhere.^20^ Moreover, lobar bleeds have been associated with greater absolute perihematomal edema volume than deep, spreading more widely and with different morphology.^24^ We speculate that the tendency for lobar hemorrhage to take on irregular, separated or multinodular shapes results in an increased surface area to volume ratio. This may expose a greater volume of brain to pro-inflammatory stimuli leading to increased inflammation and BBB permeability.

Age was the only baseline clinical factor found to be associated with TSPO binding, where greater TSPO binding was associated with lower patient age. In contrast, increased TSPO expression measured with [^11^C](*R*)-PK11195 PET has been shown in normal aging,^25^ though one study using a different tracer ([^18^F]-FEPPA) reported no association between TSPO expression and aging.^26^ Preclinical studies have shown increased microglial activation after experimental ICH in aged rats compared to young rats,^27,28^ again contradicting our result, though it is difficult to say how translatable these findings are to our study where the age range of participants in the PET component was 46–84 years. Clinical studies investigating relationships between brain immune cell activation and age in ICH are lacking, and the potential relationship between age, microglial activation, and stroke should be further investigated.

In a multifactorial model correcting for baseline clinical factors, we found no relationship between perihematomal *K*^trans^ or *BP_ND_* and outcome, measured by mRS at 90 days. Contrary to our findings, an older study utilizing DTPA-SPECT 24–48 h after onset in acute ICH demonstrated a positive correlation between ^99m^Tc-DTPA uptake in the perihematomal region (as a ratio of the contralateral region) and mRS at 3-months.^29^ Differences may be due to different methods for quantifying BBB permeability and timing (24–48 h vs. a median 56.2 h in our study), where the present study is potentially describing a different phase of BBB pathophysiology after ICH.

Our finding that higher contralateral *K*^trans^ is associated with poor outcomes at 90 days is of considerable interest. It may be driven by a global process, and with post-hoc exploratory analysis to determine if any baseline factors were associated with contralateral *K*^trans^, we found that only more variable SBP over the first 72 h was independently associated with greater contralateral *K*^trans^. The SD of SBP over 72 h was also positively correlated with 90-day mRS in our patient sample (r = 0.396, *P = *0.02), as established in previous larger studies.^30^ Whether the well-known association between high SD SBP and higher mRS may be partly mediated through widespread blood-brain barrier disruption is an intriguing hypothesis which could have important mechanistic implications for blood pressure management after ICH, where the precise mechanism by which SBP variation worsens outcome is unknown. Variability in SBP has been shown to reduce cerebral blood flow after ICH,^31^ but no studies have investigated the relationship between BBB permeability and blood pressure variability in ICH. Further studies to test this hypothesis are certainly required.

The strengths of our study are the use of advanced imaging techniques during separate, dedicated research scans to quantify BBB permeability and TSPO binding in a cohort of patients representative of clinical practice, with a large median ICH volume of 26.2 ml. Similar studies are often dominated by patients with small ICH volumes due to the difficulty associated with performing advanced imaging in unwell patients, making it challenging to extrapolate results. We have been able to minimize this bias. Only patients deemed medically stable were able to participate in the PET imaging component of the study, however we found no difference between ICH volumes, GCS, or NIHSS scores between the MR only and PET groups. Patients received prospective assessment of long-term outcome, with a face-to-face research assessment at 90 days, ensuring accuracy of the 90-day mRS and only 2 patients (6%) were lost to follow-up.

Our study also has some limitations. Due to practicalities of scanner access and patient acceptability, MRI scans took place 1–4 days after onset, while PET scans took place 2–8 days after onset. Reassuringly, we found no association between *K*^trans^ or *BP_ND_* and time from onset to scan. For patients having both imaging modalities, the interval between scans ranged from 1–5 days, with a median of 2.8 days between scans. Given practical constraints, greater precision of scan timing would likely have been prohibitively challenging. Nevertheless, these remain relatively large time intervals in terms of the evolution of secondary injury after ICH. The inflammatory response after ICH is dynamic and is thought to perhaps enhance recovery after 72 h; some PET data were collected as late as 8 days after onset, and this may have influenced some of our findings relating to immune cell activation and outcome. Simultaneous imaging using a combined PET-MR scanner would be beneficial for future studies and could overcome some of the logistical challenges associated with this study. There are further logistical and ethical challenges associated with serial PET imaging, as well as serial dosing of gadolinium contrast agents. However, the simultaneous spatiotemporal characterization of both the neuroinflammatory and BBB response after ICH would be undeniably useful. Perhaps with the continued development of tracer-free imaging methods for the measurement of BBB permeability and immune cell activation, this will become possible.

## Supplemental Material

sj-pdf-1-jcb-10.1177_0271678X241274685 - Supplemental material for Neuroinflammation and blood-brain barrier breakdown in acute, clinical intracerebral hemorrhage Supplemental material, sj-pdf-1-jcb-10.1177_0271678X241274685 for Neuroinflammation and blood-brain barrier breakdown in acute, clinical intracerebral hemorrhage by Olivia A Jones, Saffwan Mohamed, Rainer Hinz, Alastair Paterson, Oluwaseun A Sobowale, Ben R Dickie, Laura M Parkes and Adrian R Parry-Jones in Journal of Cerebral Blood Flow & Metabolism
