# Supplementary material for: Neuroinflammation and blood-brain barrier breakdown in acute, clinical intracerebral hemorrhage
Source: J Cereb Blood Flow Metab. 2024 Oct 3;45(2):233–43. doi: 10.1177/0271678X241274685 (PMC11563506; doi:10.1177/0271678X241274685)
Supplement: sj-pdf-1-jcb-10.1177_0271678X241274685 - Supplemental material for Neuroinflammation and blood-brain barrier breakdown in acute, clinical intracerebral hemorrhage [file sj-pdf-1-jcb-10.1177_0271678X241274685.pdf]

Supplementary Materials

Supplementary Methods

DCE-MRI Analysis

DCE-MRI quantities, processes and models described herein are OSIPI CAPLEX compliant.[1] CAPLEX definitions can be accessed by clicking on quantity, process r model hyperlinks. The dynamic series of post-contrast T_1-weighted images were aligned using the first dynamic image as a reference using the co-registration function in SPM12. The registered dynamic series of T_1-weighted images were used to generate a signal time course, $S_t(t)$, which was converted to a concentration time course, $C_t(t)$, using the pre-contrast (native) R_1 map (R_{10}) and the longitudinal relaxivity of the contrast agent, which was assumed to be 3.4 $s^{-1}mM^{-1}$. A Patlak model (equation 1) of contrast agent uptake was used:

$$C_t(t) = K^{\mathrm{trans}} \int_0^t C_p(t')dt' + v_p C_p(t) \qquad (1)$$

The model was fit to the concentration time-course C(t) on a voxel-wise basis using constrained non-linear least-squares minimisation (lsqcurvefit in Matlab) for 3 parameters: the contrast agent volume transfer constant across the BBB, K^{trans}, the blood plasma volume, v_p, and T_0 (where T_0 is the offset time between $C_t(t)$ and $C_p(t)$).

Constraints on the fitted parameters were as follows: between -0.001 min^{-1} 0.1 min^{-1} for K^{trans}, between 0 and (1 – hematocrit) for v_p, and between -20 and 20 s for T_0. Though unphysiological, the negative lower bound for K^{trans} is necessary to avoid positive bias in K^{trans} values, which may be very close to zero in healthy tissue. The negative lower bound on T_0 allows for the possibility that the regional blood circulation may occur later than the sagittal sinus peak concentration.

The plasma concentration, C_p, was derived using equation 2:

$$C_p(t) = C_b(t)/(1 - hematocrit) \qquad (2)$$

where the blood concentration, C_b, was derived from a vascular input function. The input function was generated using a region of approximately 50 voxels drawn manually within the superior sagittal sinus on the last dynamic image in the DCE-MRI series. The superior sagittal

sinus has been recommended by consensus guidelines as it is a large vessel with clear post-contrast enhancement, leading to ease of delineation without partial volume effects, and without inflow effects.[2] The mean signal within this region was extracted for each dynamic to generate the concentration-time curve, C_b. Measured values for hematocrit were used for each patient.

Peripheral Inflammatory Markers

At the time of MR and PET scanning, respectively, approximately 6 ml of venous blood was taken. Blood for assays of inflammatory markers was collected into ethylenediaminetetra-acetic acid (EDTA)-treated tubes. Blood samples were centrifuged at 2000 g, 4°C within six-hours of venepuncture. The plasma was stored at −70°C until blinded analysis was undertaken. Measurement of IL-6 was by enzyme-linked immunosorbent assay (ELISA), using PeliPair anti-IL-6 antibodies (M9316; Sanquin, Amsterdam, the Netherlands) essentially as per the Sanquin protocol, but with the following differences, 5% horse serum (B9433; Sigma, Poole, UK) and 1% mouse serum (M5096, Sigma) are added to the HPE used for the 1h incubation with samples, standards and controls, the detection antibody is used at half the suggested concentration and ZyMax streptavidin–horseradish peroxidise conjugate (Zymed Laboratories, San Francisco, CA, USA) in tris buffer, 1% bovine serum albumin and 0·1% Tween 20 (Sigma, Poole, UK) is used.

Plasma CRP was measured in a single-plex competitive assay using Luminex bead technology (Luminex, Austin, TX, USA), 10% horse serum, 5% bovine serum, and 1% mouse serum diluent in tris-buffered saline as a dilutent. Bio-Plex magnetic COOH beads (Bio-Rad Laboratories, Hemel Hempstead, UK) were coupled to Biodesign anti-CRP monoclonal antibody (cat: M86842M, clone C2. The competitor was CRP (P100-0; SCIPAC, Sittingbourne, UK) biotinylated with Pierce EZ-Link Sulfo-NHS-LC-LC-Biotin (Pierce, Rockford, IL, USA). Binding of biotinylated CRP was assessed, following addition of R-Phycoerythrin Streptavidin (Jackson ImmunoResearch Laboratories Inc, Stratech, Newmarket, UK; Cat: 016-110-084), using a Bio-Plex 200 system.

Supplementary Results

Post-hoc analysis of relationships with contralateral K^{trans}

Supplementary Table 1. Association between contralateral K^{trans} and clinical factors thought to influence relationships with outcome.

Measure	Contralateral K^{trans}	
	β coefficient (95% CI)	*p*
Location (Deep)	0.094 (-0.214 to 0.333)	0.6
Time since onset (days)	-0.109 (-0.009 to 0.005)	0.5
Age (Years)	-0.078 (-0.12 to 0.008)	0.7
ICH Volume (mm^3)	0.292 (-0.047 to 0.222)	0.2
Mean SBP (mm Hg)	0.049 (-0.009 to 0.011)	0.2
SD SBP (mm Hg)	0.507 (0.001 to 0.035)	**0.04***

Abbreviations: ICH = intracerebral hemorrhage; SBP = systolic blood pressure.

*P < 0.05

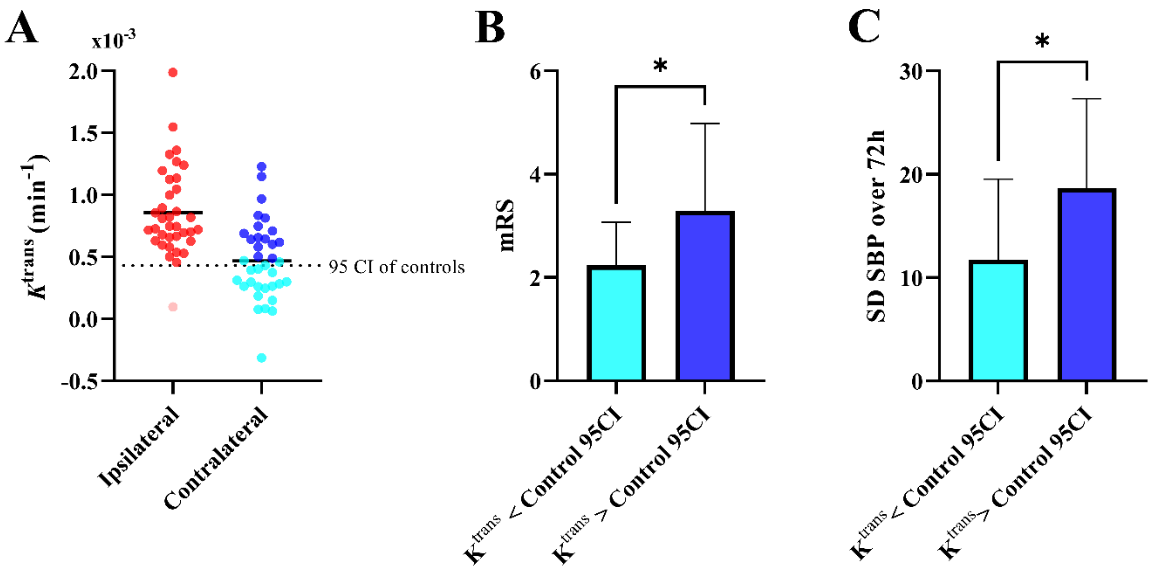

Supplementary Figure 1. **(A)** Individual perihematomal (ipsilateral) and contralateral K^{trans} values plotted with a line representing the 95% confidence interval (95CI) of grey matter control data from another study.[3] Values above the 95% CI are shown in dark blue (contralateral) and dark red (ipsilateral). **(B)** Bar chart comparing mRS score for those with

contralateral K^{trans} greater than the 95CI of controls (dark blue) and less than the 95CI of controls (light blue). **(C)** Bar chart comparing SD SBP values for those with contralateral K^{trans} greater than the 95CI of controls (dark blue) and less than the 95CI of controls (light blue). Error bars shown are standard deviation on the mean. Statistical significance is indicated for unpaired t-tests, with * representing P < 0.05.

References

1. Dickie BR, Ahmed Z, Arvidsson J, et al. A community-endorsed open-source lexicon for contrast agent-based perfusion MRI: A consensus guidelines report from the ISMRM Open Science Initiative for Perfusion Imaging (OSIPI). *Magn Reson Med*. Published online October 13, 2023. doi:10.1002/mrm.29840

2. Thrippleton M, Backes W, Sourbron S, et al. Quantifying blood-brain barrier leakage in small vessel disease: Review and consensus recommendations. *Alzheimer's & Dementia*. 2019;15. doi:10.1016/j.jalz.2019.01.013

3. Al-Bachari S, Naish JH, Parker GJM, Emsley HCA, Parkes LM. Blood-Brain Barrier Leakage Is Increased in Parkinson's Disease. *Front Physiol*. 2020;11:593026. doi:10.3389/fphys.2020.593026
